# Estimation of gingival crevicular fluid oxidative stress markers in school-aged children and teenagers with insufficient sleep

**DOI:** 10.1186/s12903-022-02642-z

**Published:** 2022-12-18

**Authors:** Qianwen Yin, Chao Liu, Han Bao, Size Li, Zhuwei Huang, Deao Gu, Liping Xiong, Leiying Miao

**Affiliations:** 1 Department of Orthodontics, Nanjing Stomatological Hospital, Medical School, Nanjing University, No. 30, Central Road, Nanjing, 210000 People’s Republic of China; 2 Department of Cariology and Endodontics, Nanjing Stomatological Hospital, Medical School, Nanjing University, No. 30, Central Road, Nanjing, 210000 People’s Republic of China

**Keywords:** Oxidative stress, Insufficient sleep, Gingival crevicular fluid, Reactive oxygen species, Superoxide glutathione

## Abstract

**Background:**

Sleep is crucial for survival. Sleep deprivation causes ROS accumulation and, consequently, oxidative stress. The goal of the study was to evaluate gingival crevicular fluid (GCF) levels of the oxidative stress status hydrogen peroxide (H_2_O_2_), superoxide glutathione (GSH), and cellular oxidative damage marker malondialdehyde (MDA) in school-aged children and teenagers with insufficient sleep.

**Methods:**

This study investigated sleep duration in 80 participants from two different developmental stages: school-aged children (6–13 years) and teenagers (14–17 years). GCF samples were obtained from all individuals, and samples were investigated to detect H_2_O_2_, GSH, and MDA levels using the micro method.

**Results:**

Results reveal that GCF MDA and H_2_O_2_ in school-age children and teenagers with insufficient sleep were significantly higher than in children with sufficient sleep. GCF GSH with insufficient sleep was insignificantly lower than in children with sufficient sleep. There was no significant difference between school-age and teenage populations.

**Conclusion:**

Sleep deprivation causes increased levels of oxidative stress in gingival crevicular fluid, and adequate sleep is essential for maintaining redox balance.

## Background

Insufficient sleep leads to dysfunction of cognition, immunity, metabolism, and circulatory systems [1–4]. Numerous studies have linked sleep deprivation to serious health problems [5, 6], and sleep restriction can cause premature mortality in model organisms, including rats, flies, and dogs [7, 8]. Adequate sleep in early life development is necessary for normal brain function, as the brain maturation of children and adolescents occurs at a critical time after birth [9]. Sufficient sleep duration is the basis for children's sleep health. Sleep deprivation could harm academic performance and increase the risk of cognitive deficits and mood disorders [10, 11]. Concerning studies of diseases associated with sleep and dental practice, Wieczorek et al. concluded that sleep duration is not linked to sleep bruxism [12]. Smardz et al. concluded that there was no statistically significant correlation between the intensity of sleep bruxism and stress, but also stated that the effect of psycho-emotional state on the severity of sleep bruxism needs to be further explored [13]. Yatani et al. reported a significant association between sleep disturbance and painful TMD [14].

Low and medium levels of reactive nitrogen (RNS) and reactive oxygen species (ROS) play essential roles in performing different cellular functions and are essential for optimal cell health [9]. When RNS or ROS exceeds the antioxidant capacity of cells, oxidative stress will occur, resulting in cellular damage by oxidizing lipids, proteins, and DNA [15]. ROS produced in the body mainly include hydroxyl radicals (^·^OH), superoxide radicals (^·^O_2_^−^), hydrogen peroxide (H_2_O_2_), singlet oxygen, and lipid hydroperoxides [16]. One proposed function of sleep is to supply the brain with antioxidants, which may be an adaptive response to sleep deprivation, which may cause oxidative stress [17]. Animal models have also revealed a relationship between oxidative stress and sleep deprivation [18]. An animal experiment has shown that insufficient sleep can lead to death by accumulating reactive oxygen species in the gut [19]. Human blood transcriptome analysis revealed that the biological processes encoded by sleep restriction genes are most affected by oxidative stress responses and cellular responses to reactive oxygen species [20].

So far, the previous research on the harmful effects of sleep loss on organs has focused on the main large organs of organisms as the heart and brain [21], and there is a lack of research on the effects on the oral environment. Whether oxidative stress caused by sleep deprivation causes elevated ROS in the oral environment. Previous studies of redox state in the oral environment have focused on saliva [22, 23]. Researchers are increasingly focusing on GCF as a diagnostic tool for analyzing oral diseases and treatment outcomes. GCF is exposed to fewer oral environmental stressors than saliva, which results in GCF being a peculiar oral fluid (plasma exudate). GCF is a suitable oral matrix for noninvasive sampling in this assay [24].

Due to the biochemical instability of ROS, it is difficult to direct measurements and quantify their content. Nevertheless, ROS-induced tissue destruction can be assessed by analyzing markers of oxidative stress, such as changes in antioxidant enzyme activities [25]. Glutathione system (GSH) are among the most important members of the antioxidant defense system and is used to assess the antioxidant capacity of an organism [21]. H_2_O_2_ and superoxide have been the research focus on ROS biology in recent years. Superoxide is easily and rapidly converted into H_2_O_2_ in cells, so that we will measure H_2_O_2_ as the primary ROS member [26]. Malondialdehyde (MDA) is a biomarker of oxidative stress injury in tissues [23, 27]. We hypothesized that insufficient sleep's oxidative stress state in the body might affect GCF oxidative stress levels. Therefore, this study aimed to detect crevicular fluid oxidative stress levels among school-aged children and teenagers with insufficient sleep and to investigate the correlation with oxidative stress in vivo.

## Methods

### Patients

Eighty participants were recruited from the Department of Nanjing Stomatological Hospital in China for one year, from September 2021 to August 2022. The study has obtained ethical clearance from the Nanjing Stomatological Hospital Institutional Ethics Committee (Approval N. NJSH-2021NL-91). The individuals were introduced to the purpose of the research and planned procedures, and written consent was taken from each subject. This study used a cross-sectional study and convenience sampling method. We used a questionnaire to collect information on gender, age, sleep duration, tooth brushing time, and the number of times. A single operator examined all participants' basic periodontal parameters, including plaque, debris and calculus index, which were assessed and recorded. Moreover, gingival crevicular fluid samples were collected from each patient for subsequent detection of oxidative stress levels. The inclusion and exclusion criteria are shown in Table 1.

**Table 1:** Participant’ recruited criteria

The inclusion criteria	(i) Participants with no systemic diseases or treatments
	(ii) Reliable and cooperative patients
	(iii) 6–17 years old (school-aged children/teenagers)
	(iv)Subjects with insufficient sleep (sleep duration of fewer than 7 h) and sufficient sleep (sleep duration of more than 7 h)
	(v)No smoking history
	(vi) No obesity
The exclusion criteria	(i) Addictive participants on alcohol/ cigarettes/drug/
	(ii) Obesity
	(iii) ongoing orthodontic treatment
	(iv) Unreliable and uncooperative patients

### Study design

Participants were divided into two groups according to their age. Participants' daily sleep duration was recorded for a week, including weekday and weekend sleep duration [28]. The National Sleep Foundation (NSF) consensus report states that nine to eleven hours is recommended for school-aged children aged 6–13. In comparison, eight to ten hours is suggested for those teenagers aged 14 to 17 years. Sleep duration of fewer than 7 h was defined as insufficient sleep [29]. All participants in the study were evaluated for the basic periodontal parameter, the whole-mouth plaque index (PI) (excluding third molars), and the OHI-S index (simplified oral hygiene index) [30, 31]. Oral hygiene maintenance, such as tooth brushing time and times, was also recorded for all participants.

### Collection of gingival crevicular fluid

Maxillary incisors were selected to collect GCF samples from each participant. Supragingival plaque on the tooth surface was removed before sampling. The tooth surface was dried and cotton rolls were moisture-barrier saliva to facilitate collection of GCFs. GCF sampling was collected with absorbent paper points (Gapadent, China). Carefully insert the paper points into the hole and let it sit for 30 s to avoid mechanical damage. It was discarded if the GCF collection filter strips were contaminated with blood. The paper points with GCF were immediately immersed in sterile polypropylene tubes with 500μL of phosphate-buffered saline (PBS, pH 7.2). The samples were stored at – 80 °C until analyzed. All samples were collected in triplicate.

### Measurement of oxidative stress markers in GCF

The samples were thawed at room temperature and eluted using centrifugation at 10,000×g for 10 min before removing the Periopaper strips [32]. Supernatants were used to determine oxidative stress levels using a commercially available kit (Solarbio, China).

### Malondialdehyde measurements

MDA can be condensed with thiobarbituric acid (TBA) to generate a brownish-red trimethyloxazole-2,4-dione with a maximum absorption wavelength of 532 nm. Estimates the amount of MDA in a sample after performing colorimetry. Results expressed as nmol/ml.

### Glutathione measurements

Glutathione can react with DTNB to produce TNB and glutathione disulfide. TNB is a yellow product with maximum light absorption at 412 nm. Its absorbance is directly proportional to GSH content. Results expressed as µg/ml.

### Hydrogen peroxide measurements

H_2_O_2_ and titanium sulfate produce yellow titanium peroxide complex with characteristic absorption at 415 nm. Results expressed as µmol/ml.

### Statistical analysis

The sample size used G*Power software to calculated with an effect size of 0.4. In each group, 13 participants were required to have 80% chance (beta error) of detecting a significant difference (two-sided 5% level). The normality of data distribution uses the Shapiro–Wilk test to assess. Continuous data were summarized as mean ± SD. Compare the continuous data variance using the two-way analysis between the two groups. Data were analyzed by SPSS 25.0 software package. Values of *p* < 0.05 were considered statistically significant.

## Results

We illustrate the analyzed study groups' demographic characteristics, sleep duration, and baseline periodontal parameters. The average age of all subjects was 12.98 ± 2.74 years old, and there was no significant difference in age between groups. There were 36 males (45%) and 44 females (55%), and there was no significant difference in gender between groups. The mean sleep duration of school-aged children and teenagers was 7.02 ± 1.21 h. In the school-aged children Group, the mean sleep duration was 8.25 ± 0.68 h in the sufficient sleep group and 6.32 ± 0.24 h in the insufficient sleep group. The mean sleep duration was significantly lower in the insufficient sleep group than in the sufficient sleep group (*p* < 0.01). In the teenager Group, the mean sleep duration was 7.7 ± 1.11 h in the sufficient sleep group and 5.8 ± 0.47 h in the insufficient sleep group, and the difference was statistically significant (*p* < 0.01). Periodontal baseline parameters and oral hygiene practices were not statistically different (Table 2).

**Table 2:** Descriptive data for the study participants

Parameters	All cases (n = 80)	School aged children Group (n = 40)			Teenagers Group (n = 40)		
		Sufficient sleep (n = 20)	Insufficient sleep (n = 20)	p-Value	Sufficient sleep (n = 20)	Insufficient sleep (n = 20)	p-Value
Age (years), mean ± SD	12.98 ± 2.74	10.75 ± 2.0	10.8 ± 2.12	0.939^a^	15.25 ± 1.16	15.10 ± 0.97	0.66^a^
(min–max)	(6–17)	(7–13)	(6–13)		(14–17)	(14–17)	
*Gender*							
Male *n* (%)	36 (45%)	8 (40%)	9 (45%)	1^b^	10 (50%)	9 (45%)	1^b^
Female *n* (%)	44 (55%)	12 (60%)	11 (55%)		10 (50%)	11 (55%)	
Sleep duration,	7.02 ± 1.21	8.25 ± 0.68	6.32 ± 0.24	< 0.01^a^	7.7 ± 1.11	5.8 ± 0.47	< 0.01^a^
*Mean* ± *SD*							
PI	1.13 ± 0.17	1.01 ± 0.15	1.08 ± 0.16	0.135^a^	1.19 ± 0.12	1.23 ± 0.13	0.372^a^
OHI-S	3.29 ± 1.66	2.35 ± 1.69	2.9 ± 1.37	0.266^a^	3.60 ± 1.60	4.30 ± 1.34	0.142^a^
Number of tooth brushing	2.58 ± 0.61	2.55 ± 0.69	2.6 ± 0.6	0.807^a^	2.5 ± 0.69	2.65 ± 0.49	0.432^a^
Brushing time	3.14 ± 0.61	3.1 ± 0.79	3.0 ± 0.73	0.679^a^	3.25 ± 0.44	3.2 ± 0.41	0.714^a^

SD—standard deviation; ^a^ Student’s *t*-test; ^b^ Correction for continuity

### Gingival crevicular fluid oxidative stress markers

Among school-aged children, the sleep-deprived group showed higher H_2_O_2_ levels, significantly different from the sleep-sufficient group (*P* < 0.01). H_2_O_2_ levels were highest in the sleep deprivation group among the adolescent population, significantly different from H_2_O_2_ levels in the sleep sufficiency group (*P* < 0.01). Sleep duration significantly affected H_2_O_2_ levels (*p* < 0.01). However, the effect of age on H_2_O_2_ levels was no significant difference (*p* = 0.61), and the interaction between sleep duration and age had no significant difference on H_2_O_2_ levels (*p* = 0.83) (Tables 3 and 4).

**Table 3:** Intergroup comparison of sleep duration and oxidative stress level parameters (independent samples test)

Groups	Parameters	N	H_2_O_2_ (µmol/mL)		GSH (µg/mL)		MDA (nmol/mL)	
			Mean ± SD	*P*	Mean ± SD	*P*	Mean ± SD	*P*
School-aged children	Sufficient sleep	20	0.29 ± 0.11	< 0.01**	25.47 ± 2.77	0.827	0.16 ± 0.02	0.013*
	Insufficient sleep	20	0.57 ± 0.19		24.62 ± 2.69		0.26 ± 0.03	
Teenagers	Sufficient sleep	20	0.31 ± 0.12	< 0.01**	26.20 ± 2.84	0.419	0.18 ± 0.03	0.027*
	Insufficient sleep	20	0.58 ± 0.15		23.09 ± 2.53		0.28 ± 0.03	

*p < 0.05, **p < 0.01

**Table 4:** Effect of sleep duration and age group on H_2_O_2_ levels (two-way ANOVA)

Parameters	*DF*	SS	*MS*	*F*	*P*
Age	1	0.006	0.006	0.26	0.61
Sleep duration	1	1.55	1.55	72.57	< 0.01*
Age* Sleep duration	1	0.001	0.001	0.05	0.83

*p < 0.05

GSH levels were below in the school-aged children with sleep deprivation than in the sleep sufficiency group; however, the data were not significantly difference (*p* = 0.83). GSH levels were lowest in the adolescent with sleep deprivation; however, the data were not significantly different (*p* = 0.42). Sleep duration had no significant effect on GSH levels (*p* = 0.47). Age also had no significant effect on GSH levels (*p* = 0.88). The interaction between sleep duration and age was no significant difference in GSH levels (*p* = 0.68) (Tables 3 and 5).

**Table 5:** Effect of sleep duration and age group on GSH levels (two -way ANOVA)

Parameters	*DF*	SS	*MS*	*F*	*P*
Age	1	3.20	3.20	0.02	0.88
sleep duration	1	78.53	78.53	0.53	0.47
Age* Sleep duration	1	25.47	25.47	0.17	0.68

The sleep-deprived school-aged children showed higher MDA levels. Compared with the sufficient sleep group, the difference was statistically significant (*p* = 0.01). MDA levels were higher in the teenager’s sleep-deprived group; the difference was statistically significant compared with the sufficient sleep group (*p* = 0.03). Sleep duration had a significant effect on MDA level (*p* < 0.01), but age had no significant effect on MDA level (*p* = 0.56). The interaction between sleep duration and age had no significant effect on MDA level (*p* = 0.9) (Tables 3 and 6).

**Table 6:** Effect of sleep duration and age group on MDA levels (two -way ANOVA)

Parameters	*DF*	SS	*MS*	*F*	*P*
Age	1	0.005	0.005	0.34	0.56
sleep duration	1	0.19	0.19	12.05	0.001*
Age* Sleep duration	1	0.0002	0.0002	0.02	0.90

*p < 0.05

## Discussion

It's important to note that the National Sleep Foundation stresses that some people may be able to sleep longer or less than recommended without ill effects. However, those who sleep much shorter than the average long-term may develop serious health problems and well-being [29]. Animal models of chronic sleep deprivation suggest increased oxidative stress and free radical production [33]. Obstructive sleep apnea (OSA) is a frequent sleep disorder. Mukherjee et al. have demonstrated that genetic factors directly impact OSA susceptibility [34]. Wieckiewicz et al. showed that the *HTR2A rs2770304* polymorphism might be associated with an association between bruxism (SB) and OSA [35]. The purpose of the study was to detect the levels of oxidative stress markers, H_2_O_2_, GSH, and MDA, in school-aged children and teenagers with insufficient sleep and compare the oxidative stress level severity within two groups of the population.

The Centers for Disease Control and Prevention (CDC) survey data show that more than half of children do not get enough sleep during school [36]. Insufficient sleep also exists in Chinese children [37]. Accumulating evidence suggests that sleep loss in pediatric populations has a significant harm impact and that a reduction in sleep duration impairs emotional functioning and cognitive in developing children [38, 39]. Studies have shown that even a few days of moderate sleep restriction can impair mood and cognitive function [40]. Children may not recover from sleep restriction as quickly as adults [41]. Therefore, the subjects of this study focused on children and adolescents. The National Sleep Foundation recommends less than 7 h as a non-recommended sleep duration for school-aged children and teenagers, so we divide adequate sleep and lack of sleep by 7 h [29].

Free radicals are involved in many normal biological processes. High concentrations of free radicals may cause tissue damage. Some enzymatic antioxidants, such as reduced glutathione (GSH), protect tissues from oxidative damage caused by free radicals generated by various metabolic events [42]. Glutathione is considered a major free radical scavenger, reflecting the extent to which tissues are challenged by oxidation stress [21]. Lipids are one of the most damaging components of ROS to cells. Oxidative stress caused by redox imbalance leads to lipid peroxidation [43]. Malondialdehyde (MDA) is a commonly used indicator to measure oxidative lipid damage caused by free radicals [39]. It has been shown that serum MDA levels are increased in systemic diseases such as tuberculosis [44]. Oxygenated derivatives vary widely in reactivity and half-life. H_2_O_2_ is one of the organisms' most common reactive oxygen species molecules and a significant signal for mitochondrial ROS. In addition, it is a relatively weak oxidant but rather stable to facilitate detection [45]. Therefore, we selected GSH, MDA, and H_2_O_2_ as test indicators in this study.

Periodontal disease has been reported to affect general health and increase the risk of cardiovascular disease, diabetes and other diseases [46, 47]. It has also been suggested that *P. gingivalis* in periodontal pockets may not significantly affect the deterioration of the heart valve [48]. Białowąs et al. concluded that periodontal therapy seems to have a salutary effect on the activity of rheumatoid arthritis [49]. Prosthetics in the mouth also affects GCF contents. Heboyan et al. concluded that polymorphonuclear neutrophils (PMNs) in GCF after repair with different fixed restorations significantly differed from before repair [50]. GCFs are biological fluids in the gingival sulcus, derived from plasma. They are defined as transudate or exudate [51]. Therefore, disease diagnosis by analysis of GCF is convenient for individuals. At the same time, GCF is known to be an essential diagnostic material and, unless invasive, contains host cell products (cytokines, antibodies, enzymes), plasma-derived molecules, subgingival microbial products, and tissue destruction products [52]. Tóthová et al. suggested that higher oxidative stress and lower antioxidant status could be detected in saliva, plasma, and GCF of patients with periodontitis [53]. The physiology of GCF may be influenced by gingival inflammation, orthodontic activation, general health, and individual differences. Herein, we strictly followed the inclusion and exclusion criteria (excluding possible interfering factors such as ongoing orthodontic treatment and history of systemic diseases individual), and selected a large sample size. Previous studies have not focused on the effects of sleep deprivation on oxidative stress in GCF, and this study is innovative. It helps identify the harm of sleep deprivation to local oral tissues and endangering large organs. Although sleep deprivation is not a causative factor in periodontal tissue inflammation, it may become a risk factor and aggravate the present inflammation.

The results showed that the levels of MDA and H_2_O_2_ in teenagers and school-aged children with insufficient sleep were significantly higher than in sufficient sleep. These results suggest that sleep deprivation increases the production of oxidative stress products in the gingival crevicular fluid. GSH levels were below in the insufficient sleep group compared to in the sleep sufficiency group. These results indicate that the antioxidant strength of gingival crevicular fluid is relatively weakened during sleep deprivation. Sleep deprivation may cause oxidative/antioxidant imbalance and oxidative stress in gingival crevicular fluid. Still, there was no significant difference between different populations. The study reveals that insufficient sleep can induce oxidative stress in the gingival crevicular fluid by directly decreasing antioxidant levels and increasing free radical production, such as glutathione. To some extent, insufficient sleep may prevent the occurrence of processes that normally occur during sleep. The statistical results of plaque index showed no difference, suggesting that the difference in oxidative stress level in the gingival crevicular fluid was influenced by sleep duration. It may further aggravate oxidative stress levels in the gingival crevicular fluid of patients with periodontal tissue disease, aggravating inflammatory conditions. Literature suggests a direct link between chronic periodontitis and levels of oxidative stress-related biomarkers in GCF [52]. Wadie et al. concluded that the severity of periodontitis is related to the expression of cytokines such as TGF-β and vimentin [54].

This study has some limitations. A limitation of this study is the limited age span of the study population, and adults and older adults with insufficient sleep were not included in the study. If this population is included, the effect of elevated ROS in GCF on periodontal inflammation can be observed. Second, this survey was cross-sectional and the causal relationship between sleep duration and gingival crevice fluid oxidative stress in school-age and adolescents cannot be determined, which still needs to be further verified by prospective cohort studies.

## Conclusion

Sleep deprivation affects general health, and oral health is also essential. We analyzed oxidative stress biomarkers in gingival crevicular fluid in sufficient sleep versus insufficient sleep groups in a pediatric and adolescent population, and MDA, H_2_O_2_, and GSH were good oxidative stress markers. Our study suggests that sleep deprivation increases oxidative stress markers in gingival crevicular fluid and impacts oral redox balance. However, no association was found between age stage and oxidative stress levels in gingival crevicular fluid.

## Data Availability

All data generated or analysed during this study are included in this published article.

## References

[1] Krueger JM, Frank MG, Wisor JP, Roy S (2016). Sleep function: toward elucidating an enigma. Sleep Med Rev.

[2] Irwin MR (2019). Sleep and inflammation: partners in sickness and in health. Nat Rev Immunol.

[3] McAlpine CS, Kiss MG, Rattik S, He S, Vassalli A, Valet C (2019). Sleep modulates haematopoiesis and protects against atherosclerosis. Nature.

[4] Tobaldini E, Fiorelli EM, Solbiati M, Costantino G, Nobili L, Montano N (2019). Short sleep duration and cardiometabolic risk: from pathophysiology to clinical evidence. Nat Rev Cardiol.

[5] Medic G, Wille M, Hemels ME (2017). Short- and long-term health consequences of sleep disruption. Nat Sci Sleep.

[6] Chattu VK, Manzar MD, Kumary S, Burman D, Spence DW, Pandi-Perumal SR (2018). The global problem of insufficient sleep and its serious public health implications. Healthcare (Basel)..

[7] Shaw PJ, Tononi G, Greenspan RJ, Robinson DF (2002). Stress response genes protect against lethal effects of sleep deprivation in Drosophila. Nature.

[8] Bentivoglio M, Grassi-Zucconi G (1997). The pioneering experimental studies on sleep deprivation. Sleep.

[9] Atrooz F, Salim S (2020). Sleep deprivation, oxidative stress and inflammation. Adv Protein Chem Struct Biol.

[10] Dewald JF, Meijer AM, Oort FJ, Kerkhof GA, Bogels SM (2010). The influence of sleep quality, sleep duration and sleepiness on school performance in children and adolescents: a meta-analytic review. Sleep Med Rev.

[11] Gringras P, Green D, Wright B, Rush C, Sparrowhawk M, Pratt K (2014). Weighted blankets and sleep in autistic children–a randomized controlled trial. Pediatrics.

[12] Wieczorek T, Wieckiewicz M, Smardz J, Wojakowska A, Michalek-Zrabkowska M, Mazur G (2020). Sleep structure in sleep bruxism: a polysomnographic study including bruxism activity phenotypes across sleep stages. J Sleep Res.

[13] Smardz J, Martynowicz H, Wojakowska A, Michalek-Zrabkowska M, Mazur G, Wieckiewicz M (2019). Correlation between sleep bruxism, stress, and depression-a polysomnographic study. J Clin Med..

[14] Yatani H, Studts J, Cordova M, Carlson CR, Okeson JP (2002). Comparison of sleep quality and clinical and psychologic characteristics in patients with temporomandibular disorders. J Orofac Pain.

[15] Betteridge DJ (2000). What is oxidative stress?. Metabolism.

[16] Di Meo S, Reed TT, Venditti P, Victor VM (2016). Role of ROS and RNS Sources in Physiological and Pathological Conditions. Oxid Med Cell Longev.

[17] Reimund E (1994). The free radical flux theory of sleep. Med Hypotheses.

[18] Villafuerte G, Miguel-Puga A, Rodriguez EM, Machado S, Manjarrez E, Arias-Carrion O (2015). Sleep deprivation and oxidative stress in animal models: a systematic review. Oxid Med Cell Longev.

[19] Vaccaro A, Kaplan Dor Y, Nambara K, Pollina EA, Lin C, Greenberg ME (2020). Sleep loss can cause death through accumulation of reactive oxygen species in the gut. Cell..

[20] Moller-Levet CS, Archer SN, Bucca G, Laing EE, Slak A, Kabiljo R (2013). Effects of insufficient sleep on circadian rhythmicity and expression amplitude of the human blood transcriptome. Proc Natl Acad Sci USA.

[21] Everson CA, Laatsch CD, Hogg N (2005). Antioxidant defense responses to sleep loss and sleep recovery. Am J Physiol Regul Integr Comp Physiol.

[22] Takane M, Sugano N, Ezawa T, Uchiyama T, Ito K (2005). A marker of oxidative stress in saliva: association with periodontally-involved teeth of a hopeless prognosis. J Oral Sci.

[23] Khanna R, Thapa PB, Khanna HD, Khanna S, Khanna AK, Shukla HS (2005). Lipid peroxidation and antioxidant enzyme status in oral carcinoma patients. Kathmandu Univ Med J (KUMJ).

[24] Khurshid Z, Mali M, Naseem M, Najeeb S, Zafar MS (2017). Human gingival crevicular fluids (GCF) proteomics: an overview. Dent J (Basel).

[25] Becerik S, Ozturk VO, Celec P, Kamodyova N, Atilla G, Emingil G (2017). Gingival crevicular fluid and plasma oxidative stress markers and TGM-2 levels in chronic periodontitis. Arch Oral Biol.

[26] Gough DR, Cotter TG (2011). Hydrogen peroxide: a Jekyll and Hyde signalling molecule. Cell Death Dis.

[27] Tsikas D (2017). Assessment of lipid peroxidation by measuring malondialdehyde (MDA) and relatives in biological samples: Analytical and biological challenges. Anal Biochem.

[28] Li H, Ji CY, Zong XN, Zhang YQ (2009). Body mass index growth curves for Chinese children and adolescents aged 0 to 18 years. Zhonghua Er Ke Za Zhi.

[29] Hirshkowitz M, Whiton K, Albert SM, Alessi C, Bruni O, DonCarlos L (2015). National sleep foundation's sleep time duration recommendations: methodology and results summary. Sleep Health.

[30] Loe H (1967). The gingival index, the plaque index and the retention index systems. J Periodontol..

[31] Greene JC, Vermillion JR (1964). The simplified oral hygiene index. J Am Dent Assoc.

[32] Chandra RV, Sailaja S, Reddy AA (2017). Estimation of tissue and crevicular fluid oxidative stress marker in premenopausal, perimenopausal and postmenopausal women with chronic periodontitis. Gerodontology.

[33] McEwen BS, Karatsoreos IN (2015). Sleep deprivation and circadian disruption: stress, allostasis, and allostatic load. Sleep Med Clin.

[34] Mukherjee S, Saxena R, Palmer LJ (2018). The genetics of obstructive sleep apnoea. Respirology.

[35] Wieckiewicz M, Bogunia-Kubik K, Mazur G, Danel D, Smardz J, Wojakowska A (2020). Genetic basis of sleep bruxism and sleep apnea-response to a medical puzzle. Sci Rep.

[36] Wheaton AG, Jones SE, Cooper AC, Croft JB (2018). Short sleep duration among middle school and high school students—United States, 2015. MMWR-Morbid Mortal Wkly Rep.

[37] Wu J, Wu H, Wang J, Guo L, Deng XQ, Lu CY (2015). Associations between sleep duration and overweight/obesity: results from 66,817 Chinese adolescents. Sci Rep-UK.

[38] Vriend JL, Davidson FD, Corkum PV, Rusak B, Chambers CT, McLaughlin EN (2013). Manipulating sleep duration alters emotional functioning and cognitive performance in children. J Pediatr Psychol.

[39] Talbot LS, McGlinchey EL, Kaplan KA, Dahl RE, Harvey AG (2010). Sleep deprivation in adolescents and adults: changes in affect. Emotion.

[40] Vriend J, Davidson F, Rusak B, Corkum P (2015). Emotional and cognitive impact of sleep restriction in children. Sleep Med Clin.

[41] Carskadon MA, Harvey K, Dement WC (1981). Sleep loss in young adolescents. Sleep.

[42] Day BJ (2009). Catalase and glutathione peroxidase mimics. Biochem Pharmacol.

[43] Kara A, Akman S, Ozkanlar S, Tozoglu U, Kalkan Y, Canakci CF (2013). Immune modulatory and antioxidant effects of melatonin in experimental periodontitis in rats. Free Radic Biol Med.

[44] Khalili J, Biloklytska HF (2008). Salivary malondialdehyde levels in clinically healthy and periodontal diseased individuals. Oral Dis.

[45] Knaus UG (2021). Oxidants in physiological processes. Handb Exp Pharmacol.

[46] Sanz M, Marco Del Castillo A, Jepsen S, Gonzalez-Juanatey JR, D'Aiuto F, Bouchard P (2020). Periodontitis and cardiovascular diseases: consensus report. J Clin Periodontol.

[47] Bendek MJ, Canedo-Marroquin G, Realini O, Retamal IN, Hernandez M, Hoare A (2021). Periodontitis and gestational diabetes mellitus: a potential inflammatory vicious cycle. Int J Mol Sci..

[48] Radwan-Oczko M, Jaworski A, Dus I, Plonek T, Szulc M, Kustrzycki W (2014). Porphyromonas gingivalis in periodontal pockets and heart valves. Virulence.

[49] Bialowas K, Radwan-Oczko M, Dus-Ilnicka I, Korman L, Swierkot J (2020). Periodontal disease and influence of periodontal treatment on disease activity in patients with rheumatoid arthritis and spondyloarthritis. Rheumatol Int.

[50] Heboyan A, Syed AUY, Rokaya D, Cooper PR, Manrikyan M, Markaryan M (2020). Cytomorphometric analysis of inflammation dynamics in the periodontium following the use of fixed dental prostheses. Molecules.

[51] Goodson JM (2000). Gingival crevice fluid flow. Periodontol.

[52] Chen M, Cai W, Zhao S, Shi L, Chen Y, Li X (2019). Oxidative stress-related biomarkers in saliva and gingival crevicular fluid associated with chronic periodontitis: a systematic review and meta-analysis. J Clin Periodontol.

[53] Tothova L, Celec P (2017). Oxidative stress and antioxidants in the diagnosis and therapy of periodontitis. Front Physiol.

[54] Wadie KW, Bashir MH, Abbass MMS (2021). Epithelial-mesenchymal transition in gingival tissues from chronic periodontitis patients: a case-control study. Dent Med Probl.

